# Relative effectiveness of high-dose versus standard-dose influenza vaccine against hospitalizations and mortality according to Charlson Comorbidity Index: A post-hoc analysis of the DANFLU-1 randomized trial

**DOI:** 10.1007/s10096-026-05408-5

**Published:** 2026-01-29

**Authors:** Katrine Feldballe Bernholm, Niklas Dyrby Johansen, Caroline Espersen, Daniel Modin, Kira Janstrup Hyldekær, Joshua Nealon, Sandrine Samson, Matthew M. Loiacono, Rebecca C. Harris, Carsten Schade Larsen, Anne Marie Reimer Jensen, Nino Emanuel Landler, Signe Tellerup Nielsen, Lene Russell, Theis Skovsgaard Itenov, Brian L. Claggett, Scott D. Solomon, Martin J. Landray, Gunnar H. Gislason, Lars Køber, Pradeesh Sivapalan, Jens Ulrik Stæhr Jensen, Tor Biering-Sørensen

**Affiliations:** 1 Center for Translational Cardiology and Pragmatic Randomized Trials, Department of Biomedical Sciences, Faculty of Health and Medical Sciences, University of Copenhagen, Copenhagen, Denmark; 2 Cardiovascular Non-Invasive Imaging Research Laboratory, Department of Cardiology, Copenhagen University Hospital - Herlev and Gentofte, Copenhagen, Denmark; 3 Sanofi, Lyon, France; 4 Sanofi, PA Swiftwater, USA; 5 Sanofi, Singapore; 6 Department of Clinical Medicine, Department of Infectious Diseases, Aarhus University Hospital, Aarhus, Denmark; 7 Department of Intensive Care, Copenhagen University Hospital - Herlev and Gentofte, Copenhagen, Denmark; 8 Department of Anaesthesia and Intensive Care, Copenhagen University Hospital – Bispebjerg and Frederiksberg, Copenhagen, Denmark; 9 Cardiovascular Division, Brigham and Women’s Hospital, Harvard Medical School, Boston, MA USA; 10 Clinical Trial Service Unit and Epidemiological Studies Unit, Nuffield Department of Population Health, University of Oxford, Oxford, UK; 11 Department of Clinical Medicine, Faculty of Health and Medical Sciences, University of Copenhagen, Copenhagen, Denmark; 12 The Danish Heart Foundation, Copenhagen, Denmark; 13 The National Institute of Public Health, University of Southern Denmark, Copenhagen, Denmark; 14 Department of Cardiology, Copenhagen University Hospital – Rigshospitalet , Copenhagen, Denmark; 15 Copenhagen Respiratory Research, Department of Medicine, Copenhagen University Hospital – Herlev and Gentofte, Copenhagen, Denmark; 16 Steno Diabetes Center Copenhagen, Copenhagen, Denmark

**Keywords:** Influenza vaccine, Comorbidity, Charlson Comorbidity Index, Pragmatic randomized controlled trial, Hospitalizations, Mortality

## Abstract

**Purpose:**

The DANFLU-1 trial suggested lower incidence of hospitalizations for pneumonia and influenza, respiratory disease and all-cause mortality among older adults receiving high-dose (HD-IV) versus standard-dose (SD-IV) influenza vaccine. This study assessed the relative effectiveness of HD-IV versus SD-IV according to comorbidity in elderly individuals.

**Methods:**

This was a post-hoc analysis of the DANFLU-1 randomized controlled feasibility trial of HD-IV versus SD-IV conducted during the 2021–2022 influenza season in adults aged 65–79 years. Outcomes assessed included influenza-related, respiratory, and cardiovascular hospitalizations, and mortality. We tested for effect modification by level of the Charlson Comorbidity Index (CCI) using ICD-10 codes up to 10 years prior to randomization.

**Results:**

Of the 12,477 randomly assigned participants (mean age 71.7 ± 3.9 years, 47.1% female), 8,020 (64.3%) had CCI = 0, 3,560 (28.5%) had CCI = 1–2 and 893 (7.2%) had CCI ≥ 3. When comparing HD-IV with SD-IV, hazard ratios of hospitalizations for pneumonia and influenza were similar across CCI groups (HR [95%CI]: 0.15 [0.03–0.68] for CCI = 0, 0.36 [0.11–1.15] for CCI = 1–2, 1.00 [0.25-4.00] for CCI ≥ 3). Comparable patterns were found for hospitalizations for respiratory disease (0.46 [0.17–1.20] for CCI = 0, 0.67 [0.32–1.39] for CCI = 1–2, 0.66 [0.24–1.87] for CCI ≥ 3) and all-cause mortality (0.28 [0.09–0.86] for CCI = 0, 0.70 [0.30–1.63] for CCI = 1–2, 0.57 [0.24–1.36] for CCI ≥ 3). There was no statistical evidence of effect modification by CCI for any outcome.

**Conclusions:**

The lower incidences of clinical outcomes for HD-IV compared to SD-IV were not significantly modified by CCI. The potential benefit of HD-IV versus SD-IV may therefore be applicable regardless of comorbidity burden. Further research is required to confirm these findings.

**Supplementary Information:**

The online version contains supplementary material available at 10.1007/s10096-026-05408-5.

## Introduction

The high-dose influenza vaccine was developed to improve protection against influenza-related outcomes among older adults. Randomized trials have indicated that high-dose influenza vaccine improves vaccine efficacy, effectiveness and immune response compared with standard-dose influenza vaccine in older adults [1, 2]. These findings were supported by the DANFLU-1 trial as the incidence of hospitalization for pneumonia or influenza, hospitalization for respiratory disease, and all-cause mortality was lower among older adults (65–79 years) receiving high-dose (HD-IV) versus standard-dose (SD-IV) influenza vaccine [3]. Individuals with comorbidities are at increased risk of influenza-related complications including severe acute respiratory illness, intensive care admission, and higher hospitalization and mortality rates [4–6]. Data are sparse regarding the added benefit of high-dose versus standard-dose influenza vaccine according to level of comorbidity. The Charlson Comorbidity Index (CCI) is widely used as a weighted measure of comorbidity [7] and has been shown to predict both short- and long-term mortality [7–12]. Although CCI was originally designed to predict 1-year mortality among hospitalized patients, the index has been proven to predict relevant clinical outcomes in both in- and outpatient populations, such as major adverse cardiovascular events, respiratory failure, and intensive care unit (ICU) admission [13, 14]. Since its introduction in 1987, CCI has been continuously modified to maintain and improve applicability [8, 11]. 

HD-IV poses a promising approach to lower the risk of influenza-related complications in high-risk populations including individuals with comorbidities, and thus in this post-hoc analysis of the DANFLU-1 trial, we sought to assess the relative effectiveness of HD-IV versus SD-IV according to comorbidity as assessed by CCI in elderly individuals.

## Methods

### Study design

This was a post-hoc analysis of the DANFLU-1 trial, for which the design and main findings have previously been published [3, 15]. DANFLU-1 was a pragmatic, registry-based, open-label, active-controlled, individually randomized trial of HD-IV versus SD-IV. The trial was designed to assess feasibility of conducting large-scale pragmatic vaccine trials within the Danish healthcare system utilizing Danish national health registries as primary data source.

Eligibility criteria were (a) 65–79 years of age at time of inclusion and (b) no known allergies to study vaccines. Participants were enrolled in the trial from October 1 to November 20, 2021 and were recruited by a private vaccination provider responsible for organizing vaccination appointments under the Danish governmental vaccination program. A central site monitored the study and was responsible for data collection and safety monitoring.

### Randomization

Participants were randomized in a 1:1 ratio to HD-IV or SD-IV using central blocked randomization. As the trial was an open-label study, neither investigators, participants nor study personnel were blinded to treatment assignment. Follow-up data were retrieved passively from nationwide health registries to minimize the risk of ascertainment bias.

### Study treatment

HD-IV (Fluzone High-Dose Quadrivalent (United States and Canada)/Efluelda (Europe), Sanofi) contained 60 µg of hemagglutinin antigen for each influenza strain, whereas SD-IV (InfluvacTetra, Viatris) contained 15 µg for each strain. Both vaccine types were quadrivalent and contained the same four influenza strains as recommended by the World Health Organization for the 2021–2022 northern hemisphere influenza season.

### Data collection and baseline data

All citizens in Denmark have a unique identification number to which all public health-related and administrative data are linked on the individual-level. Data on randomization group and administered vaccine were collected by the vaccination provider and transferred to the central trial site. All other trial data were obtained from the Danish nationwide registries by the central site investigators. The Danish nationwide registries contain data on all hospital contacts (both in- and outpatient contacts), procedures, deaths and redeemed prescriptions [16–18]. 

Information on baseline comorbidities and medication use were obtained using prespecified definitions based on the International Classification of Disease, 10th edition (ICD-10) and Anatomical Therapeutic Chemical (ATC) classification codes. Baseline comorbidities were assessed within 10 years prior to enrolment date.

### Charlson Comorbidity Index

The Charlson Comorbidity Index (CCI) is calculated as the sum of weights of a list of relevant comorbidities at baseline and up to 10 years prior for each participant. CCI was computed according to ICD-10 codes for these relevant comorbidities [7]. Supplemental Material Table S1 lists the CCI variables, corresponding ICD-10 codes and the Charlson weight of each variable used to compute the CCI score. Participants were divided into three groups based on the level of comorbidities categorized as low (CCI = 0), medium (CCI = 1–2) and high (CCI ≥ 3).

To account for variation in disease definitions and frequently used modifications of CCI, we performed sensitivity analysis on the effect of HD-IV versus SD-IV on hospitalization for pneumonia or influenza and all-cause mortality according to respective CCI scores. The included scores were: CCI modified according to the DANFLU-1 trial (CCI_mod_), age-adjusted CCI (CCI_age_) and CCI modified by Quan (CCI_quan_). CCI_mod_ included the CCI ICD-10 disease codes, and additionally disease codes defined in the DANFLU-1 trial [15]. For example, participants who claimed ≥ 1 prescription of medication in the Anatomical Therapeutic Chemical (ATC) category A10 (“Drugs used in diabetes”) scored 1 point in CCI_mod_ category “Diabetes without complications”. This approach has been applied to the CCI score previously [19, 20]. All additional disease codes for CCI_mod_ are listed in Supplemental Material, Table S1. CCI_age_ was computed according to the age-adjusted CCI proposed by *Charlson et al.* [21] as a re-evaluation of the original index. CCI_age_ is computed according to the age of each participant by adding 1 point for each decade from 60 years of age to the original CCI. CC_quan_ is computed according to the modification by *Quan et al.* [8] in which weights of the different comorbidities in CCI were reassigned based on administrative data from 6 countries. Components and weights for the different CCI scores are listed in Supplemental Material, Table S1.

### Outcomes

We evaluated the following prespecified clinical outcomes in relation to CCI; hospitalizations for (1) influenza or pneumonia, (2) respiratory disease, (3) cardio-respiratory disease, (4) cardiovascular disease, and (5) all causes, and (6) all-cause mortality. In addition, we evaluated (7) admission to the ICU as an additional exploratory outcome for the purpose of this present post-hoc analysis. All outcomes besides all-cause mortality were evaluated as both first and recurrent events. The follow-up period for clinical outcomes started 14 days after vaccination and ended May 31st, 2022.

### Statistical analysis

All outcome analyses were intention-to-treat. Baseline characteristics are stratified by CCI group. Continuous variables are presented as mean (standard deviation (SD)) and median (interquartile range (IQR)) for normally and non-normally distributed variables, respectively. Categorical variables are presented as absolute numbers and percentages. The main effect for all outcomes except for ICU admission has previously been reported by *Johansen et al.* [3], in which the relative vaccine effectiveness was calculated as 1 minus the relative risk of the specified outcomes with corresponding confidence intervals calculated using the Clopper-Pearson method [3]. In the current study we used Cox proportional hazards models to analyze the effect of HD-IV versus SD-IV on time-to-first event according to CCI group. For corresponding analyses using recurrent events, we calculated incidence rate ratios (IRR) using negative binominal regression models. Follow-up time was included as an offset in the negative binomial regression models. We performed likelihood ratio tests to evaluate the interaction between CCI as a grouped variable and treatment allocation in both Cox and negative binomial regression analyses to test for effect modification. Furthermore, we evaluated interaction between CCI as a continuous variable and treatment allocation. Hospitalization rates per 100 person-years according to CCI group were compared using unadjusted negative binomial regression models. A p-value < 0.05 was considered statistically significant. We used SAS Software, version 9.4 (SAS Institute), Stata MP, version 17.0 (StataCorp), and R, version 4.2.2 (R Foundation for Statistical Computing) for the statistical analysis.

## Results

A total of 12,477 participants were included in the final analysis set of the DANFLU-1 trial. Of these, 6,245 were randomized to HD-IV and 6,232 were randomized to SD-IV. Mean age was 71.7 ± 3.9 years, 47.1% were women, 20.4% had chronic cardiovascular disease, 6.8% had chronic lung disease, 10.9% had cancer and 9.3% had diabetes (Table 1). Baseline characteristics for the DANFLU-1 trial population have previously been published in full [3]. We were unable to obtain registry data for 4 individuals (2 in each randomization group). Median CCI for the population was 0 (IQR 0–1) and overall range of CCI was 0–11. In total, 8,020 participants had CCI = 0, 3,557 participants had CCI = 1–2 and 892 participants had CCI ≥ 3. Participants with CCI ≥ 3 were older (72.9 years vs. 71.4 years) and more frequently male (64.2% vs. 50.5%) compared to participants with CCI = 0 (Table 1). All assessed baseline comorbidities were more frequent with higher CCI group, and level of CCI was balanced across randomized groups (Table 1).

**Table 1:** Baseline characteristics according to Charlson Comorbidity Index

	All *N* = 12,477*	CCI = 0 *N* = 8,020 (64.3%)	CCI = 1–2 *N* = 3,560 (28.5%)	CCI ≥ 3 *N* = 893 (7.2%)
Age, years	71.7 ± 3.9	71.4 ± 3.9	72.1 ± 4.0	72.9 ± 3.9
Female sex	5,877 (47.1%)	3,966 (49.5%)	1,590 (44.7%)	320 (35.8%)
Chronic lung disease	850 (6.8%)	23 (0.3%)	609 (17.1%)	218 (24.4%)
Chronic obstructive pulmonary disease	417 (3.3%)	0 (0.0%)	282 (7.9%)	135 (15.1%)
Chronic cardiovascular disease	2,540 (20.4%)	944 (11.8%)	1204 (33.8%)	392 (43.8%)
Ischemic heart disease	962 (7.7%)	283 (3.5%)	477 (13.4%)	202 (22.6%)
Heart failure	275 (2.2%)	0 (0%)	161 (4.5%)	114 (12.8%)
Atrial fibrillation	878 (7.0%)	388 (4.8%)	375 (10.5%)	115 (12.9%)
Hypertension	6,469 (51.9%)	3,538 (44.1%)	2,263 (63.6%)	668 (74.8%)
Diabetes	1,162 (9.3%)	326 (4.1%)	546 (15.3%)	290 (32.5%)
Cerebrovascular disease	456 (3.7%)	0 (0%)	327 (9.2%)	129 (14.5%)
Cancer	1,363 (10.9%)	0 (0%)	856 (24.0%)	507 (56.8%)
Immunodeficiency	483 (3.9%)	179 (2.2%)	214 (6.0%)	90 (10.1%)
CCI	0 (0–1)	0 (0–0)	1 (1–2)	3 (3–4)
HD-IV	6,245 (50.1%)	4,024 (50.2%)	1,773 (49.8%)	446 (49.9%)

Data are presented as mean ± SD, median (IQR) or n (%). CCI, Charlson Comorbidity Index; HD-IV, high-dose influenza vaccine. *We could not obtain registry data for 4 participants (2 in each randomization group)

**Fig. 1:**
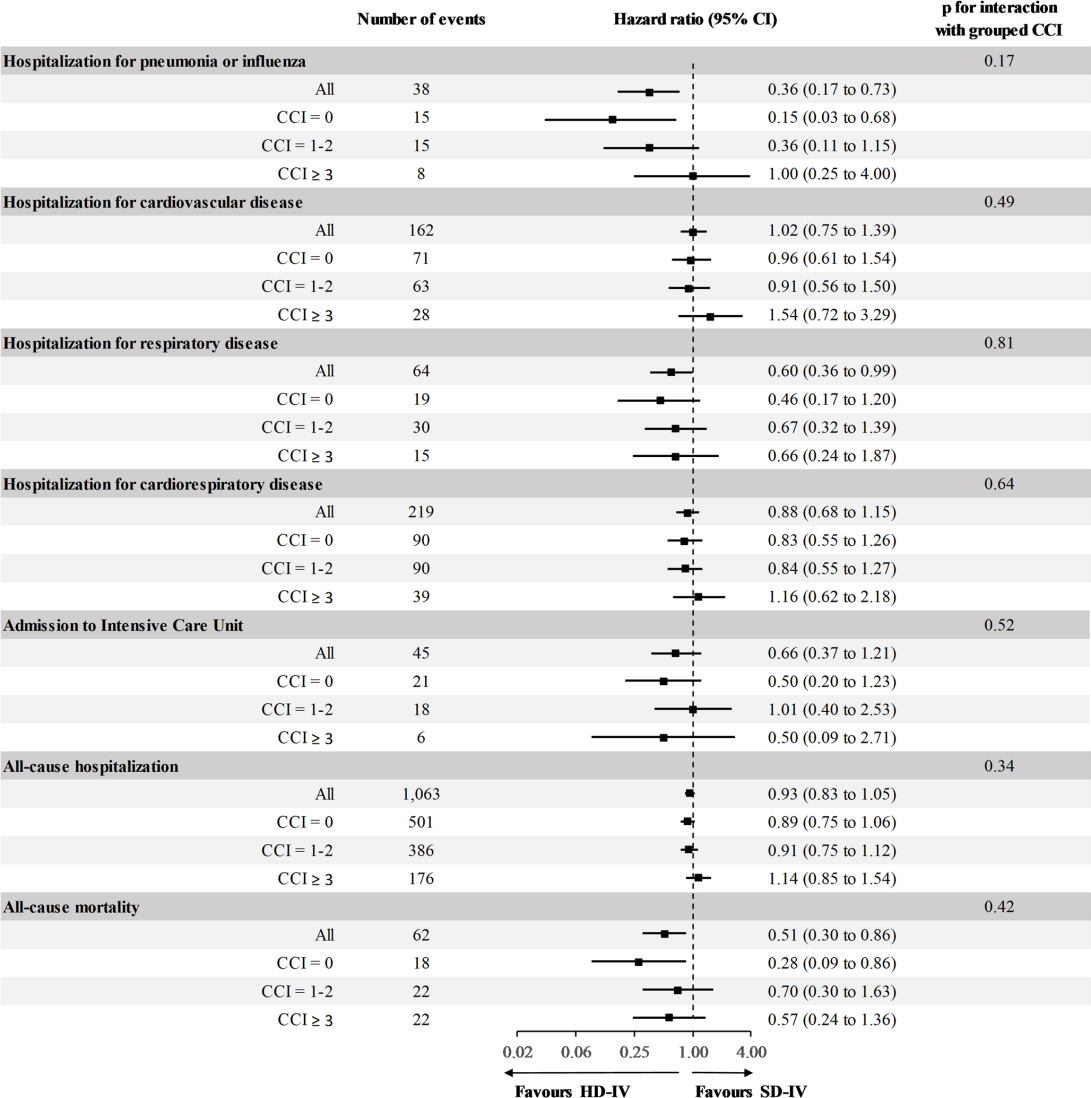
Relative effectiveness of HD-IV compared with SD-IV against time to first events according to Charlson Comorbidity Index (CCI). Hazard ratios with 95% confidence intervals were derived from Cox proportional hazard models. HD-IV, high-dose influenza vaccine; SD-IV, standard-dose influenza vaccine; CCI, Charlson Comorbidity Index

The median follow-up time was 237 days (IQR 232–239). In time to first event analysis, a total of 38 (0.3%) participants were hospitalized with influenza or pneumonia, 64 (0.5%) for respiratory disease, 1,063 (8.5%) for all-cause hospitalization, 45 (0.4%) participants were admitted to the ICU, and 62 (0.5%) participants died. Overall, HD-IV versus SD-IV was associated with a lower incidence of hospitalization for pneumonia or influenza, hospitalization for respiratory disease, and all-cause mortality. There was no evidence that this was significantly modified by CCI as both grouped (p_interaction_ with grouped CCI: 0.17, 0.81 and 0.42, respectively) (Fig. 1) and CCI as continuous variable (p_interaction_ with continuous CCI: 0.34, 1.00 and 0.96, respectively).

**Fig. 2:**
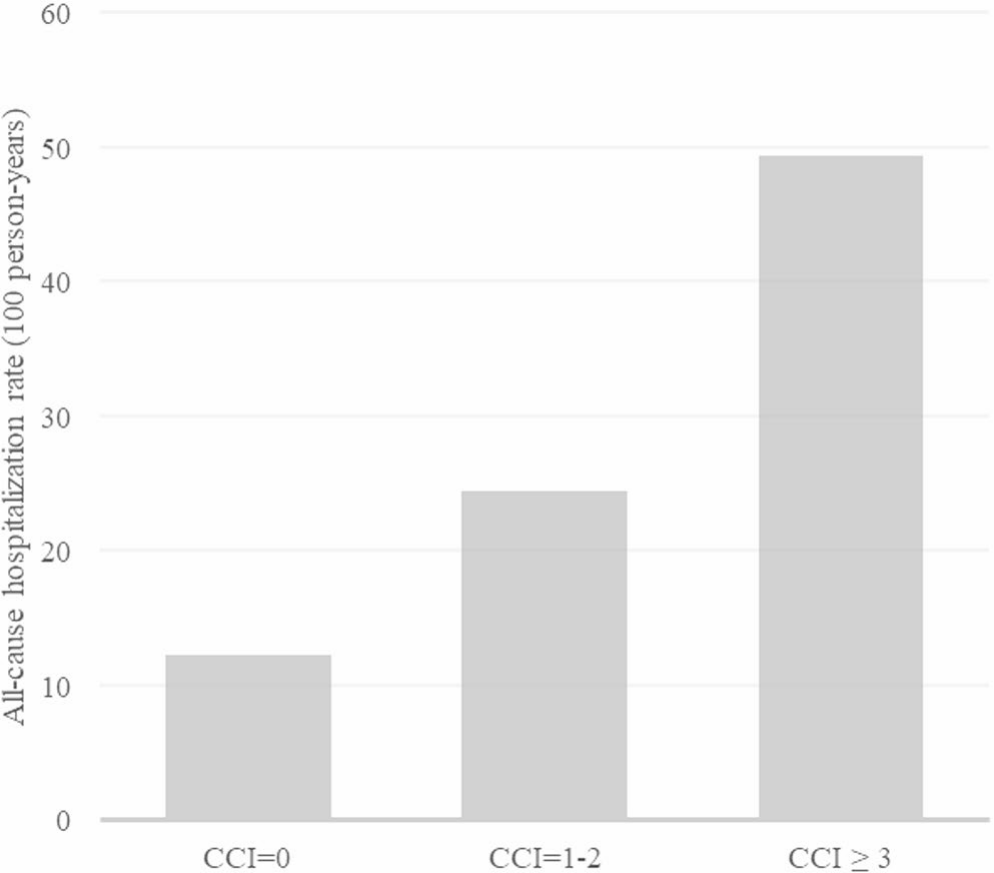
All-cause hospitalization rates per 100 person-years according to Charlson Comorbidity Index (CCI)

**Fig. 3:**
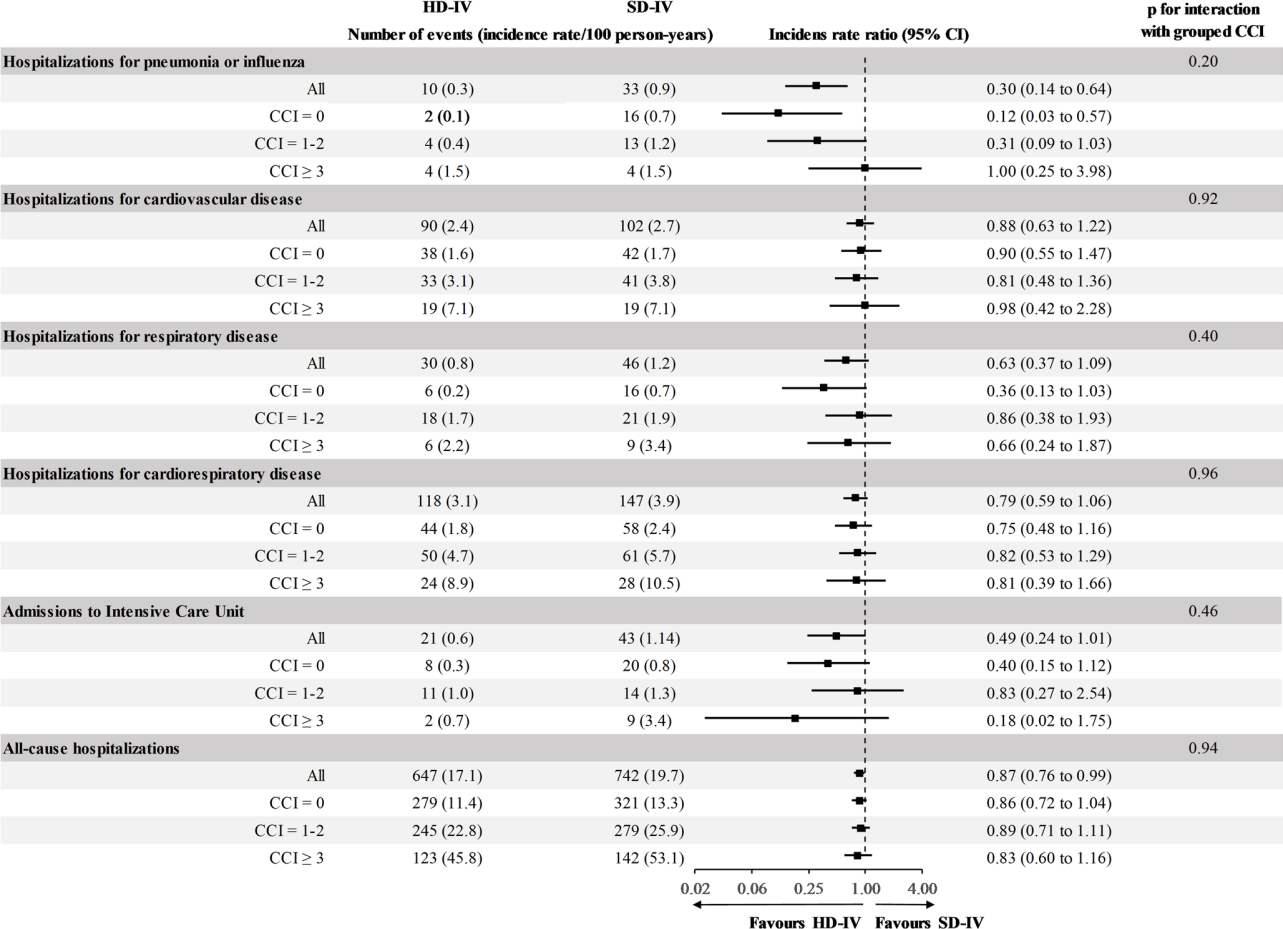
Relative effectiveness of HD-IV compared with SD-IV against recurrent events according to Charlson Comorbidity Index (CCI). Incidence rate ratios with 95% confidence intervals were derived from negative binomial regression models. HD-IV, high-dose influenza vaccine; SD-IV, standard-dose influenza vaccine; CCI, Charlson Comorbidity Index

In recurrent events analysis, we observed a total of 43 hospitalizations for pneumonia or influenza, 76 for respiratory disease, 64 admissions to the ICU and 1,389 all-cause hospitalizations. All-cause hospitalization rates per 100 person years during follow-up were higher with increasing CCI group (12 for CCI = 0 vs. 24 for CCI = 1–2 and 49 for CCI ≥ 3, *p* < 0.001 for all comparisons between groups, Fig. 2). Overall, HD-IV versus SD-IV was associated with lower incidence rates of hospitalizations for pneumonia or influenza and all-cause hospitalizations, and this association was not significantly modified by CCI as both grouped (p_interaction_ with grouped CCI: 0.20 and 0.94, respectively) (Fig. 3) and CCI as continuous variable (p_interaction_ with continuous CCI: 0.16 and 0.74, respectively).

In sensitivity analysis of three different modifications of CCI, we found a redistribution of participants in the respective CCI groups (Supplemental Material, Tabel S2). Numerically fewer participants had CCI = 0 for both CCI_mod_ and CCI_age_ compared to the original CCI (7,607 and 0 versus 8,020, respectively) and more participants had CCI = 0 for CCI_quan_ (9,101) (Supplemental Material, Table S2). Median CCI values were 0 (IQR 0–1) for both CCI_mod_ and CCI_quan_, and 2 (IQR 1–3) for CCI_age_. CCI_age_ and CCI_quan_ significantly modified the association of HD-IV versus SD-IV with the risk of hospitalizations for pneumonia or influenza (p_interaction_ with grouped CCI: 0.01 and 0.02, respectively) (Supplemental Material, Figure S1). CCI_mod_ did not modify this association (p_interaction_ with grouped CCI: 0.22). None of the assessed CCI scores modified the effect of HD-IV compared with SD-IV for all-cause mortality (p_interaction_ with grouped CCI: 0.39, 0.23 and 0.30 for CCI_mod_, CCI_age_ and CCI_quan_, respectively) (Supplemental Material, Figure S1).

## Discussion

In this post-hoc analysis of a randomized, pragmatic feasibility trial, HD-IV versus SD-IV was associated with a lower risk of hospitalization for pneumonia and influenza, respiratory disease, and all-cause mortality irrespective of CCI. These findings suggest that HD-IV may be similarly effective and beneficial for influenza-related outcomes regardless of comorbidity burden.

Patients with comorbidities are at increased risk of adverse outcomes associated with influenza [4–6]. Increasing chronological age is associated with elevated comorbidity burden and due to immunosenescence in older individuals, a lower immune response to influenza vaccination is observed in this population [22, 23]. This immune system degradation enhances development of chronic diseases and these conditions then contributes to further immunosenescence including inflammaging, cellular senescence and decreased T cell activity [22]. Although increasing chronological age is considered the culprit of immunosenescence and hence the altered immune response against both the vaccine and infection, number and severity of comorbidities in itself has been reported as independent risk factor of influenza-related hospital admissions in adults ≥ 18 years [24]. To prevent adverse events and to increase health span in patients with comorbidity, it is important to ensure high influenza vaccine efficacy and effectiveness. High-dose influenza vaccine has been shown to increase serological antibody response in patients ≥ 65 years of age regardless of comorbidity [25]. In the INVESTED trial [26], similar increase in serological response was found in patients with cardiovascular comorbidity, although no association between serological response and risk of adverse outcomes was found. Furthermore, no significant effect of high-dose versus standard-dose influenza vaccine was found. Included patients had either heart failure or recent acute myocardial infarction and at least one additional risk factor (e.g., diabetes, renal impairment etc.). As such, the INVESTED population had a high level of significant comorbidity of which the disease trajectories may not have been modifiable by the added effect of high-dose versus standard-dose influenza vaccine. In a pragmatic, cluster-randomized single-blind trial, *Gravenstein et al.* found high-dose versus standard-dose influenza vaccine decreased the risk of influenza-related hospitalizations in older nursing home residents [27]. This population also had a high burden of comorbidity including Alzheimer’s disease and related dementias (64% of study population), diabetes (34% of study population) and chronic respiratory disease (20% of study population). The results in these two studies of populations with high prevalence of comorbidity, could indicate that different comorbidities (e.g., cardiovascular vs. neurological) may have various disease trajectories and some might be more modifiable than others by preventive modalities including the high-dose influenza vaccine. In the present study HD-IV versus SD-IV was associated with lower incidence of hospitalization for pneumonia or influenza, respiratory disease, and all-cause mortality, regardless of comorbidity status as assessed by CCI, suggesting HD-IV to have similarly beneficial effects regardless of degree of comorbidity.

The Charlson Comorbidity Index was developed to predict 1-year mortality in hospitalized patients by assigning comorbidities weights from 1 to 6, to account for the differential contribution of each comorbidity to mortality risk [7]. The index is now widely used as a valid measure of comorbidity [28]. In the main analysis in the present post-hoc study, we used CCI as proposed by *Charlson et al.* [7] with adaption by *Deyo et al.* [11] to be computed according to ICD-codes. Validation of CCI has subsequently been performed in cohorts consisting of hospitalized patients to ensure availability of data, and hence feasibility to calculate the index [8, 19, 28–30]. In studies by *Crooks et al.* [31] and *Pylväläinen et al.* [20], CCI was validated through national registries in general populations in England and Finland, respectively. Furthermore, *Thygesen et al.* [32] found that codes for CCI from the Danish National Patient Registry had a 98% positive predictive value, stating a high reliability when assessing CCI through Danish nationwide registries.

Numerous modifications have been proposed and validated to update and maintain the applicability of CCI. We performed a sensitivity analysis to address the differences and potential applicability of the most frequently used modifications of CCI. The CCI modified by *Quan et al.* [8]. (CCI_quan_) was based on data from 6 countries and reassigned weights to the assessed comorbidities, with the rationale that there had been a significant improvement in both diagnosis and treatment in certain disease categories (e.g. HIV/AIDS). CCI_quan_ has not been as broadly validated as the original CCI, especially not in the general population. The age-adjusted CCI (CCI_age_) was developed by *Charlson et al.* [21] as an index update, to constitute a simple and “crude” risk assessment score to apply in smaller studies. CCI_age_ could have a potential in general population studies as incidence of adverse event outcomes are expectedly low, although the age-adjustment may be redundant in the current study as the age range is relatively narrow. Lastly, we modified the CCI to the DANFLU-1 disease definitions (CCI_mod_) by including codes for medication prescription/redeeming. As this approach has been shown to limit underestimation of diseases not typically requiring hospitalizations, the added discriminative value to the prediction abilities of the index is conflicting [19, 20]. CCI_age_ and CCI_quan_ modified the effect of HD-IV versus SD-IV on hospitalizations for pneumonia or influenza, suggesting a potential differential effect of HD-IV versus SD-IV according to comorbidity as assessed by the respective CCI scores with the direction of decreasing relative vaccine effectiveness with increasing CCI, although this was inconsistent with the main findings of this post-hoc study. In agreement with the main analysis, none of the assessed indices modified the effect of HD-IV versus SD-IV on all-cause mortality.

This post-hoc analysis of a randomized trial has important limitations. The DANFLU-1 trial was designed as a feasibility trial and therefore not powered to assess clinical outcomes, including those reported in the current post-hoc analysis. The recently published FLUNITY-HD individually pooled analysis of the DANFLU-2 and GALFLU trials confirmed the superior protective effect of HD-IV versus SD-ID against hospitalization for pneumonia or influenza, although this was not demonstrated for all-cause mortality [33]. These findings emphasize the importance of cautious interpretation of clinical effects in trials that are not fully powered, while recognizing the role of exploratory research in generating hypotheses for future studies. We did not adjust for test multiplicity and the results of this post-hoc analysis should therefore be considered exploratory and hypothesis-generating only. Studies concerning CCI usually stratify the studied population into groups based on CCI for comprehensibility and clinical application purposes, as also performed in the current study. We found 893 (7.2%) participants to have CCI ≥ 3, indicating a relatively healthy population. This could potentially diminish the discriminative ability of CCI in the DANFLU-1 population for the purpose of this study, although the CCI has been validated in similar populations, including elderly and general populations [28]. The CCI was originally developed to predict 1-year mortality in hospitalized patients and hence we cannot with certainty state that it is an accurate or true measure of comorbidity. Furthermore, CCI is constructed as an accumulative score of ICD-10 diagnoses and does not account for advancement in disease severity except for diabetes, liver disease and cancer, where two tiers of the diseases are integrated in the score. This could lead to inaccurate risk weighting of diseases at an individual level, masking the effect of comorbidity in our analysis.

## Conclusion

In this post-hoc analysis, HD-IV was associated with lower risks and incidence rates of clinical outcomes, including hospitalizations for pneumonia and influenza, all-cause hospitalizations and mortality among older adults, and these associations were not significantly modified by CCI. The potential benefit of HD-IV versus SD-IV may therefore be applicable regardless of comorbidity burden. Further research is required to confirm these findings.

## Supplementary Information

Below is the link to the electronic supplementary material.

Supplementary Material 1 (PDF 264 KB)

## Data Availability

Data from the nationwide Danish administrative health registries are subject to Danish legislation and can only be made available to a third party under certain conditions. Please contact the corresponding author in case of any inquiries.
