# Supplementary material for: Relative effectiveness of high-dose versus standard-dose influenza vaccine against hospitalizations and mortality according to Charlson Comorbidity Index: A post-hoc analysis of the DANFLU-1 randomized trial
Source: Eur J Clin Microbiol Infect Dis. 2026 Jan 29;45(5):1323–32. doi: 10.1007/s10096-026-05408-5 (PMC13222198; doi:10.1007/s10096-026-05408-5)
Supplement: Supplementary file 1 — Supplementary Material 1 (PDF 264 KB) [file 10096_2026_5408_MOESM1_ESM.pdf]

SUPPLEMENTAL MATERIAL

Relative effectiveness of high-dose versus standard-dose influenza vaccine against hospitalizations and mortality according to Charlson Comorbidity Index: A post-hoc analysis of the DANFLU-1 randomized trial

Katrine Feldballe Bernholm, MD[1,2]; Niklas Dyrby Johansen, MD, PhD[1,2]; Caroline Espersen, MD[1,2]; Daniel Modin, MD[1,2]; Kira Hyldekær Janstrup, PhD[1,2]; Joshua Nealon, PhD[3]; Sandrine Samson, PhD[3]; Matthew M. Loiacono, PhD[4]; Rebecca C. Harris, MBioch, MSc, PhD[5]; Carsten Schade Larsen, MD, DMSc[6]; Anne Marie Reimer Jensen, MD[1,2]; Nino Emanuel Landler, MD, PhD[1,2]; Signe Tellerup Nielsen, MD, PhD[7]; Lene Russell, MD, PhD[7]; Theis Skovsgaard Itenov, MD, PhD[8]; Brian L Claggett, PhD[9]; Scott D Solomon, MD[9]; Martin J. Landray, PhD[10]; Gunnar H Gislason, MD, PhD[1,11,12,13]; Lars Køber, MD, DMSc[14,]; Pradeesh Sivapalan, MD, PhD[12,15]; Jens Ulrik Stæhr Jensen, MD, PhD[12,15]; Tor Biering-Sørensen, MD, PhD, MSc, MPH[1,2]

1: Department of Cardiology, Copenhagen University Hospital - Herlev and Gentofte, Copenhagen, Denmark

2: Center for Translational Cardiology and Pragmatic Randomized Trials, Department of Biomedical Sciences, Faculty of Health and Medical Sciences, University of Copenhagen, Denmark

3: Sanofi, Lyon, France

4: Sanofi, Swiftwater, Pennsylvania, USA

5: Sanofi, Singapore

6: Department of Clinical Medicine, Department of Infectious Diseases, Aarhus University Hospital, Aarhus, Denmark

7: Department of Intensive Care, Copenhagen University Hospital - Herlev and Gentofte, Copenhagen, Denmark

8: Department of Anaesthesia and Intensive Care, Copenhagen University Hospital – Bispebjerg and Frederiksberg, Copenhagen, Denmark

9: Cardiovascular Division, Brigham and Women's Hospital, Harvard Medical School, Boston, MA, USA

10: Clinical Trial Service Unit and Epidemiological Studies Unit, Nuffield Department of Population Health, University of Oxford, Oxford, United Kingdom

11: Department of Clinical Medicine, Faculty of Health and Medical Sciences, University of Copenhagen, Denmark

12: The Danish Heart Foundation, Copenhagen, Denmark

13: The National Institute of Public Health, University of Southern Denmark, Copenhagen, Denmark

14: Department of Cardiology, Copenhagen University Hospital – Rigshospitalet, Copenhagen, Denmark

15: Copenhagen Respiratory Research, Department of Medicine, Copenhagen University Hospital – Herlev and Gentofte, Copenhagen

Corresponding Author:

Prof. Tor Biering-Sørensen, MD, PhD, MPH, MSc

Center for Translational Cardiology and Pragmatic Randomized Trials

Department of Cardiology, Copenhagen University Hospital Herlev-Gentofte,

Gentofte Hospitalsvej 8, 2900 Hellerup, Denmark,

E-mail: tor.biering@gmail.com

Phone: +45 28 93 35 90

Supplemental Material, Table S1. Definition and codes of comorbidities in the modified Charlson Comorbidity Indices.

Comorbidity/ condition	**CCI** Disease definitions according to ICD-10 codes	**CCI_{mod}** Additional disease definitions (ICD-10 and ATC codes)	**CCI** and **CCI_{mod}** Weight	**CCI_{quan}** Weight	**CCI_{age}** Weight
Myocardial infarction	I21<br>I22<br>I252		1	0	1
Congestive Heart Failure	I099<br>I420<br>I425−I429<br>I43<br>I50<br>I110<br>I130<br>I132<br>I255<br>P290		1	2	1
Peripheral Vascular Disease	I170 I71<br>I731<br>I738<br>I739<br>I771<br>I790<br>I792<br>K551	I74	1	0	1

	K558<br>K559<br>Z958<br>Z959				
Cerebrovascular disease	G45<br>G46<br>H340<br>I60−I69		1	0	1
Dementia	F00−F03<br>F051<br>G30<br>G311	G312	1	2	1
Chronic pulmonary disease	I278<br>I279<br>J40−J41<br>J42-47<br>J40−J41<br>J60−J67<br>J684<br>J701<br>J703	A15-A16<br>D860<br>E84<br>J84<br>Z942	1	1	1
Rheumatologic disease	M05<br>M06<br>M315<br>M32−M34<br>M351<br>M353<br>M360		1	1	1

Peptic ulcer	K25−K28		1	0	1
Hemiplegia/ paraplegia	G041<br>G114<br>G801<br>G802<br>G81<br>G82<br>G830<br>G831<br>G832<br>G833<br>G834<br>G839		2	2	2
Diabetes without complications	E100<br>E101<br>E106<br>E108<br>E109<br>E110<br>E111<br>E116<br>E118<br>E119<br>E120<br>E121<br>E126<br>E128<br>E129	A10 (ATC)	1	0	1

	E130<br>E131<br>E136<br>E138<br>E139<br>E140<br>E141<br>E146<br>E148<br>E149				
Diabetes with chronic complications	E102−E105<br>E107<br>E112<br>E115<br>E117<br>E122−E125<br>E127<br>E132−E135<br>E137<br>E142−E145<br>E147		2	1	2
Mild liver disease	B18<br>K700−K703<br>K709<br>K713−K715<br>K717<br>K73<br>K74	B15-B17<br>B19<br>D684C<br>Q618<br>K75<br>K77	1	2	1

	K760<br>K762−K764<br>K768<br>K769<br>Z944				
Moderate/severe liver disease	I850<br>I859<br>I864<br>I982<br>K704<br>K711<br>K721<br>K729<br>K765−K767		3	4	3
Renal disease	I120<br>I131<br>N032−N037<br>N052−N057<br>N18<br>N19<br>N250<br>Z490−Z492<br>Z940<br>Z992	N11<br>N12<br>N14<br>N26<br>N158-N160<br>N162-N164<br>N168<br>M300<br>M313<br>M319<br>M321B<br>Q612-Q613<br>Q615<br>Q619	2	1	2

		T858-T859			
Any malignancy (tumor, leukemia, lymphoma)	C00−C26<br>C30−C34<br>C37−C41<br>C43<br>C45−C58<br>C60−C76<br>C81−C85<br>C88<br>C90−C97	C27-C29<br>C35-C36<br>C42<br>C59<br>C86-C87<br>C89	2	2	2
Metastatic solid tumor	C77−C80		6	6	6
HIV/AIDS	B20−B22<br>B24		6	4	6
Age 60-69	-	-	-	-	1
Age 70-79	-	-	-	-	2
**Maximum score for respective CCI**			29	24	31

CCI, Charlson Comorbidity Index; CCI_{mod}, Charlson Comorbidity Index modified according to diagnoses defined in DANFLU-1; CCI_{quan}, Charlson Comorbidity Index modified by Quan; CCI_{age}, age-adjusted Charlson Comorbidity Index; ICD-10, International Classification of Diseases 10th revision; ATC, Anatomical Therapeutic Chemical code.

Supplemental Material, Table S2. Characteristics of the modified Charlson Comorbidity Indices.

	All participants	CCI$_{mod}$			CCI$_{age}$			CCI$_{quan}$		
		CCI=0	CCI=1-2	CCI≥3	CCI=0	CCI=1-2	CCI≥3	CCI=0	CCI=1-2	CCI≥3
N	12,477	7,607 (61.0%)	3,851 (30.9%)	1,015 (8.1%)	0	8,693 (69.7%)	3,780 (30.3%)	9,101 (73.0%)	2,801 (22.4%)	571 (4.6%)
Age, years	71.7±3.9	71.4±3.9	72.1±4.0	72.9±3.9	-	71.1±3.9	73.1±3.7	71.5±3.9	72.2±3.9	72.7±3.8
Female sex	5,877 (47.1%)	3,809 (50.1%)	1,696 (44.0%)	371 (36.6%)	-	4,303 (49.5%)	15,573 (41.6%)	4,338 (47.7%)	1,316 (47.0%)	222 (38.9%)
HD-IV	6,245 (50.1%)	3,803 (50.0%)	1,934 (50.2%)	506 (49.9%)	-	4,343 (50.0%)	1,900 (50.3%)	4,538 (49.9%)	1,408 (50.3%)	297 (52.0%)
Respective CCI for all		0 (0-1)			2 (1-3)			0 (0-1)		

Data are presented as mean±SD, median (IQR) or n (%). CCI, Charlson Comorbidity Index; HD-IV, high-dose influenza vaccine; CCI, Charlson Comorbidity Index; CCI$_{mod}$, Charlson Comorbidity Index modified according to diagnoses defined in DANFLU-1; CCI$_{quan}$, Charlson Comorbidity Index modified by Quan; CCI$_{age}$, age-adjusted Charlson Comorbidity Index.

	HD-IV	SD-IV	Hazard ratio (95% CI)	p for interaction with grouped CCI
	No. of events/no. of participants (%)			
**Overall hospitalization for pneumonia or influenza**	10/6,233 (0.16)	28/6,202 0.45)	0.36 (0.17 to 0.73)	
**Overall all-cause mortality**	21/6,222 (0.34)	41/6,189 (0.66)	0.51 (0.30 to 0.86)	
**CCI modified with DANFLU-2 criteria**				
**Hospitalization for pneumonia or influenza**				0.22
CCI = 0	2/3,803 (0.05)	11/3,804 (0.29)	0.18 (0.04 to 0.82)	
CCI = 1-2	4/1,934 (0.21)	13/1,917 (0.68)	0.30 (0.10 to 0.93)	
CCI ≥ 3	4/506 (0.79)	4/509 (0.79)	1.00 (0.25 to 4.00)	
**All-cause mortality**				0.39
CCI = 0	4/3,803 (0.11)	14/3,804 (0.37)	0.29 (0.09 to 0.87)	
CCI = 1-2	9/1,934 (0.47)	12/1,917 (0.63)	0.74 (0.31 to 1.76)	
CCI ≥ 3	8/506 (1.58)	15/509 (2.95)	0.53 (0.23 to 1.26)	
**Age-adjusted CCI**				
**Hospitalization for pneumonia or influenza**				0.01
CCI = 0	0/0	0/0	-	
CCI = 1-2	2/4,343 (0.05)	18/4,350 (0.41)	0.11 (0.03 to 0.48)	
CCI ≥ 3	8/1,900 (0.42)	10/1,880 (0.53)	0.79 (0.31 to 2.00)	
**All-cause mortality**				0.23
CCI = 0	0/0	0/0	-	
CCI = 1-2	5/4,343 (0.12)	16/4,350 (0.37)	0.31 (0.11 to 0.85)	
CCI ≥ 3	16/1,900 (0.84)	25/1,880 (1.33)	0.63 (0.34 to 1.18)	
**CCI modified by Quan**				
**Hospitalization for pneumonia or influenza**				0.02
CCI = 0	3/4,538 (0.07)	17/4,563 (0.37)	0.18 (0.05 to 0.60)	
CCI = 1-2	3/1,408 (0.21)	10/1,393 (0.72)	0.30 (0.08 to 1.07)	
CCI ≥ 3	4/297 (1.35)	1/274 (0.36)	3.71 (0.41 to 33.16)	
**All-cause mortality**				0.30
CCI = 0	6/4,538 (0.13)	20/4,563 (0.44)	0.30 (0.12 to 0.75)	
CCI = 1-2	7/1,408 (0.50)	12/1,393 (0.86)	0.58 (0.23 to 1.46)	
CCI ≥ 3	8/297 (2.69)	9/274 (3.28)	0.82 (0.32 to 2.12)	

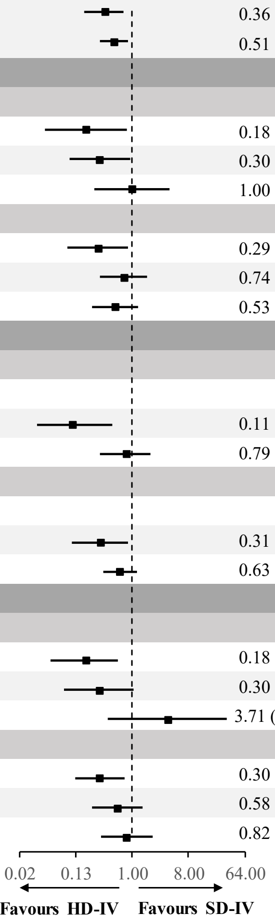

Supplemental Material, Fig. S1. Relative effectiveness of HD-IV compared with SD-IV against time to first events according to Charlson Comorbidity Indices. Hazard ratios with 95% confidence intervals were derived from Cox proportional hazard models. HD-IV, high-dose influenza vaccine; SD-IV, standard-dose influenza vaccine; CCI, Charlson Comorbidity Index.
